# Scorpion Venom Heat–Resistant Synthesized Peptide Increases Stress Resistance and Extends the Lifespan of *Caenorhabditis elegans via* the Insulin/IGF-1-Like Signal Pathway

**DOI:** 10.3389/fphar.2022.919269

**Published:** 2022-07-14

**Authors:** Ying-Zi Wang, Song-Yu Guo, Rui-Li Kong, Ao-Ran Sui, Zhen-Hua Wang, Rong-Xiao Guan, Kundu Supratik, Jie Zhao, Shao Li

**Affiliations:** ^1^ Department of Physiology, College of Basic Medical Sciences, Liaoning Provincial Key Laboratory of Cerebral Diseases, Dalian Medical University, Dalian, China; ^2^ National-Local Joint Engineering Research Center for Drug-Research and Development (R&D) of Neurodegenerative Diseases, Dalian Medical University, Dalian, China; ^3^ The Second Affiliated Hospital of Dalian Medical University, Dalian, China

**Keywords:** *Caenorhabditis elegans*, SVHRSP, stress resistance, insulin-IGF-1 like signal pathway, aging

## Abstract

Improving healthy life expectancy by targeting aging-related pathological changes has been the spotlight of geroscience. Scorpions have been used in traditional medicine in Asia and Africa for a long time. We have isolated heat-resistant peptides from scorpion venom of Buthusmartensii Karsch (SVHRP) and found that SVHRP can attenuate microglia activation and protect *Caenorhabditis elegans* (*C. elegans*) against β-amyloid toxicity. Based on the amino acid sequence of these peptides, scorpion venom heat–resistant synthesized peptide (SVHRSP) was prepared using polypeptide synthesis technology. In the present study, we used *C. elegans* as a model organism to assess the longevity-related effects and underlying molecular mechanisms of SVHRSP *in vivo*. The results showed that SVHRSP could prolong the lifespan of worms and significantly improve the age-related physiological functions of worms. SVHRSP increases the survival rate of larvae under oxidative and heat stress and decreases the level of reactive oxygen species and fat accumulation *in vivo*. Using gene-specific mutation of *C. elegans*, we found that SVHRSP-mediated prolongation of life depends on Daf-2, Daf-16, Skn-1, and Hsf-1 genes. These results indicate that the antiaging mechanism of SVHRSP in nematodes might be mediated by the insulin/insulin-like growth factor-1 signaling pathway. Meanwhile, SVHRSP could also up-regulate the expression of stress-inducing genes Hsp-16.2, Sod-3, Gei-7, and Ctl-1 associated with aging. In general, our study may have important implications for SVHRSP to promote healthy aging and provide strategies for research and development of drugs to treat age-related diseases.

## Introduction

Aging, a time-related deterioration of physiologic functions, is a global problem, occurring in all the cells, tissues, and organs (López-Otín et al., 2013). According to a report from the United Nations dated 2015, in China, the number of people over 65 years old is going to reach up to 400 million in the coming 35 years (26.9% of the total population) (Fang et al., 2015), making it one of the countries with the highest percentage of aged people in the world. Aging cannot be considered a disease, but it is one of the biggest risk factors for major age-related disorders, for instance, hypertension, diabetes, cancer, and certain neurodegenerative diseases resulting in a huge global burden (Barzilai et al., 2018). Therefore, effective components that can extend lifespan and promote healthiness have recently drawn increased attention. Hence, understanding the mechanisms responsible for lifespan extension has great potential to expose targets for drugs directed at reducing the aging process and preventing aging-associated diseases.

*Caenorhabditis elegans* (*C. elegans*) is considered an ideal model widely applied to investigate aging in organisms and explore new pharmacological targets (Ma L et al., 2018), due to its short lifespan, strong reproductive capacity, ease of genetic manipulation, and a proportion of homologous genes with humans. *C. elegans* also has more than 65% genes linked to human diseases, including conserved pathways for regulating lifespan (Stiernagle, 2006; Kenyon, 2010; Lapierre and Hansen, 2012).

Scorpions have been applied in traditional medicine for thousands of years in Asia and Africa for the treatment of epilepsy, rheumatoid arthritis, apoplexy, and chronic pain, along with other conditions (Zhou et al., 1989; Shao et al., 2007; Tajti et al., 2020; Tao et al., 2021). To date, more than 400 different peptides, toxins, or homologs have been extracted and purified from scorpion venom, and their functions have been identified, several of which have been found to practicably manage many worldwide medical problems and thus identified as drug candidates (Ortiz et al., 2015). Scorpion venom heat–resistant peptide (SVHRP) is used as a patented technology to extract a peptide mixture isolated from the Buthusmartensii Karsch venom in our laboratory. We have found that SVHRP exhibited significant inhibition of sodium channels in hippocampal neurons (Zhang et al., 2007), reduced susceptibility to epileptic seizures in rats (Chen et al., 2021), promoted neurogenesis in adult mice (Wang et al., 2014), protected against the neurotoxicity induced by β-amyloid in *C. elegans* (Zhang et al., 2016), protected against cerebral ischemia–reperfusion injury (Wang et al., 2020) and suppressed neuroinflammation and microglia activation (Wu et al., 2021). For now, the amino acid sequence of one major constituent of SVHRP has been determined using liquid chromatograph–mass spectrometry (LC–MS), and a synthetic peptide prepared in accordance with this sequence is called scorpion venom heat–resistant synthesized peptide (SVHRSP). In our previous study, we found that treatment with SVHRSP ameliorated 6-OHDA-induced neurotoxicity and neuroinflammation (Li et al., 2021). So, we hypothesize that SVHRSP has the potential to play an important role in antiaging. In this research, we used *C. elegans* as a model organism to assess the longevity-related effects and underlying molecular mechanisms of SVHRSP *in vivo*. We found that SVHRSP could extend lifespan, enhance oxidative and thermal stress resistance, and decrease reactive oxygen species (ROS) levels and fat accumulation by increasing the expression of genes that regulate stress responses and aging.

## Materials and Methods

### The Production of Scorpion Venom Heat–Resistant Synthesized Peptide

Our research group has been engaged in the research and development of Scorpion Venom drugs for many years. We extracted the scorpion venom heat-resistant peptide (SVHRP) from the scorpion of East Asia, and then further purified SVHRP and synthesized it to obtain the scorpion venom heat-resistant synthetic peptide (SVHRSP). The amino acid sequence of SVHRSP (MW = 1524.7 d) were reported in the patent (No. ZL201610645111.7) about the chemical composition and application of SVHRSP. SVHRSP used in this study was produced by GL Biochem Ltd. (Shanghai, China) with 98% purity. ddH_2_O was used as the control group in the experiment. In the previous experiments in our laboratory, we used the carbon-terminal modified peptide of SVHRSP (the same 15 amino acids as SVHRSP) for electrophysiological screening. Our results show that modified SVHRSP does not have the same effect as SVHRSP activation of sodium channels. In a similar way, modified SVHRSP did not play a role in the treatment of 6-OHDA-induced PD models of *C. elegans*. Therefore, ddH_2_O was used as the control group in the experiment.

### Strains and Breeding of *Caenorhabditis elegans*

*C. elegans* strains used in this experiment: Wild-type *C. elegans* N_2_, TJ356: Daf-16 (ZLS356:GFP) IV, CF1038: Daf-16 (MU86) I, TJ375 (HSP-16.2P:GFP), CF1553: SOD-3 (pAD76:GFP), EU1 skn-1 (zu67), PS3551 hsf-1 (sy441) I, CB1370 Daf-2 (e1370) III and CF1038 Daf-16 (mu86) I. All *C. elegans* and *E. coli* OP50 were provided by the Caenorhabditis Genetics Center (CGC); *C. elegans* used in this study are both hermaphrodite nematodes. For specific nematode culture methods, see the methods used by Brenner S (Yin et al., 2014). Nematodes feed on *E. coli* OP50, a uracil deficient strain that can grow on NGM medium without excessive growth affecting nematodes’ activity.

### Lifespan Assay

The synchronized L4 nematodes were collected, and nematodes were selected on the NGM medium with the optimal concentration of SVHRSP. Three boards were set for each concentration, with about 50–150 nematodes on each board. In order to maintain consistency of drug concentration, fresh NGM solid medium should be replaced every 2 days, the survival status of nematodes should be observed every 2 days, and the number of alive, dead, and excluded nematodes should be counted until the last day of the life of the last nematode (Solis and Petrascheck, 2011). The death criteria were: nematodes showed no signs of movement, and there was no reaction when the body was lightly touched with platinum wire; Exclusion criteria were: dry death on petri dish wall and cover; And nematodes burrowing into AGAR (Solis and Petrascheck, 2011). The life test should be repeated at least three times.

### Food Clear Assay

In order to determine the optimal concentration of SVHRSP, so as to better and effectively evaluate whether SVHRSP has an antiaging effect and influence on *C. elegans*. The purpose of the food clear assay is to establish a method for screening the optimal concentration of SVHRSP drugs for neuronal protection and effective life extension. Experimental methods: Under the condition of 20°C, the M9 solution, 10-μl SVHRSP solution, and 80-μl *E. coli* (OP50) were adjusted to 0.5–0.8 every 10–20 μl containing 30–40 adjacent nematodes and then cultured in a 96-well plate for seven consecutive days. The OD values were measured for seven consecutive days and recorded at least three times (Voisine et al., 2007).

### Lipofuscin Analysis Experiment

Lipofuscin is a highly oxidized and crosslinked protein aggregation in nematodes. It is the “marker dye” of senescence and spontaneously fluoresces blue under a fluorescence microscope (Henderson and Johnson, 2001). From 10 to 18 days, blue fluorescence could be detected in the intestinal tract of nematodes. Nematodes with different optimal drug concentrations were cultured for 5–6 days according to the feeding method in the life test. Nematodes aged from 10 to 18 days were washed with M9 at least three times and transferred to a plate containing fresh NGM. Nematodes with different SVHRSP concentrations were selected and covered with another slide in parallel to flatten the AGAR to prevent fragmentation and remove bubbles (Henderson and Johnson, 2001). Fluorescence microscopy (Leica DM4000B) (excitation wavelength 510–560 nm; emission wavelength 590–650 nm) was observed and photographed. Image J was used to analyze the spontaneous fluorescence content of lipofuscin. The lipofuscin experiment was repeated at least three times.

### Body Bend Assay

Normal *C. elegans* crawl in a sinusoidal form on a solid medium, and in the aging process, the muscles of the whole body of the *elegans* degenerate, crawl slower and bend less frequently (Ruan et al., 2016). Method: use the nematodes carrying a pole with its load pick to a single *C. elegans* to exclude OP50 NGM medium when they get used to the new environment and start crawling normally. The times of sinusoidal motion body bending in 60 s were recorded (Ruan et al., 2016). Then it was picked out, and another nematode was selected to continue the experiment; 30 nematode data were recorded in each experimental group. A new NGM medium was needed when the experimental group was replaced. Repeat the bending test at least three times.

### Pharyngeal Pumping Assay

The deglutition of nematodes is a regular contractile movement regulated using the neuromuscular system (Ryu et al., 2016). The pharyngeal pulsation of young adults is 250–300 times per minute on average. One of the obvious characteristics of senescence is the decrease in pharyngeal pulsation frequency. The specific method of the pharyngeal pump experiment is as follows: This experiment was carried out along with the lifespan experiment. Nematodes at the L4 stage were collected and selected to NGM medium with different optimal drug concentrations of SVHRSP. 10–20 nematodes from each group were randomly selected and transferred to the new NGM plate coated with the bacterial solution. The number of beats in the pharyngeal muscles of nematodes was observed under a microscope in 1 min. The pharyngeal pump test was repeated at least three times.

### Fecundity Assay

One of the most popular theories of aging is longevity and reproduction. Using a variety of advanced animal models of aging, they found a mechanism called a “tradeoff” between longevity and fertility, in which reduced or lost fertility leads to increased longevity (Gruber et al., 2007). The synchronized L4 nematodes were collected and selected to the NGM solid medium containing different concentrations of SVHRSP. Three parallel control groups were set for each concentration, with 1–2 nematodes in each group, and cultured in the constant temperature medium at 20°C. The number of oviposition of nematodes was observed every day until the reproductive cycle of nematodes was completed. The number of oviposition of nematodes in different periods of each group was counted. The oviposition experiment was repeated at least three times.

### Determination of Reactive Oxygen Specie

2, 7-dichlorodihydrofluorescein (H_2_DCF-DA) was used to detect the scavenging effect of SVHRSP on active oxygen free radicals in nematodes. Its mechanism of action is that it is oxidized by intracellular ROS into DCF to detect the fluorescence intensity and determine the free radical scavenging ability of SVHRSP (Lee et al., 2009). The specific method is as follows: the synchronized L4 nematodes were collected, and nematodes were selected on an NGM medium with different optimal drug concentrations of SVHRSP. Three boards were set for each concentration, with about 20–30 nematodes on each board. The fresh medium was changed every 2 days to ensure effective drug concentration, and *C. elegans* was cultured for 5–6 days. The supernatant was homogenized slowly on ice, centrifuged at 12000 rpm, and then added to a black 96-well plate to avoid light. At the same time, the DCFDA probe reserve solution was added, and then excited at 485 nm and emitted at 530 nm wavelength. The fluorescence value was measured with a fluorescence microplate, and the ROS experiment was repeated at least three times (Lee et al., 2009).

### Fat Accumulation Experiment

In N_2_ wild-type worms, increased production of ROS may lead to excessive fat accumulation (Hughes et al., 2007). Methods: after simultaneous culture, wild-type N_2_ nematodes were cultured in NGM medium for 96 h. The nematodes in each group were washed with M9 buffer three times, and OP50 was cleaned. An oil red O staining kit was used for lipid staining of nematodes in each group. Control and treatment worms were placed on slides, and oil red O staining was determined using fluorescein microscope (Leica DM4000B) and quantified using ImageJ software.

### Heat Shock Experiment

As nematodes age, their resistance to stress gradually decreases. The heat shock test was pretreated with 20- and 40-μM SVHRSP at 20°C for 10 days. On day 10, *C. elegans* were transferred to the new NGM petri dish and incubated at 37°C. The worms were gently touched with a platinum wire clamp every hour and marked dead if they did not respond (Strayer et al., 2003). These measurements were performed in three separate trials. Each group consisted of at least 30 animals.

### Oxidative Stress Experiment

In oxidative stress tests, 20- and 40-μM SVHRSP–treated and control nematodes were transferred to a new 6-well plate containing 30% H_2_O_2_ (Sigma, United States). The animals were monitored hourly, and when they were immobile in the liquid medium, they were recorded as dead (Pietsch et al., 2009). These measurements were performed in three separate trials. Each group consisted of at least 30 animals.

### Quantitative PCR Analysis of Gene Expression

About 800 synchronized worms were cultured with or without 20- and 40-μM SVHRSP at 20°C for 10 days. Total RNA was extracted using the RNA isolater Total RNA Extraction Reagent (Vazyme, China) and converted to cDNA. After inversion, AceQ ® QPCR SYBR Green Master Mix (low Rox premixed) kit (Vazyme, China) was used for quantitative amplification. The following primers were used: Daf-16 forward primer 5′ CGG​ATA​CCG​TAC​TCG​TGA​TGA​T 3′ and reverse primer 5′ CCA​AAC​AGC​CAC​CCA​AAT​CA, Hsp-16.2 forward primer 5′ CTG​CAG​AAT​CTC​TCC​ATC​TGA​GTC 3′ and reverse primer 5′ AGA​TTC​GAA​GCA​ACT​GCA​CC 3′, SOD-3 forward primer 5′ CCA​CCT​GTG​CAA​ACC​AGG​AT 3′ and reverse primer 5′ TGC​AAG​TAG​TAG​GCG​TGC​TC 3′, gei-7 forward primer 5′ CTG​CCA​TCT​CCG​TGG​TAT​CC 3′ and reverse primer 5′ ACC​CAT​GTT​CCA​TCG​TGT​CC 3′, ctl-1 forward primer 5′ TCG​TTC​ATG​CCA​AGG​GAG​C 3′ and reverse primer 5′ GAT​CCC​GAT​TCT​CCA​GCG​AC 3′, and act-1 forward primer 5′ CCA​GGA​ATT​GCT​GAT​CGT​ATG​CAG​AA 3′ and reverse primer 5′ TGG​AGA​GGG​AAG​CGA​GGA​TAG​A 3′.

### Daf-16 Nuclear Translocation Experiment

Under normal conditions, the insulin signaling pathway is normally activated, and a large number of phosphorylated Daf-16 is trapped in the cytoplasm, unable to enter the nucleus for transcriptional regulation. In contrast, when the insulin signaling pathway is attenuated, Daf-16 is not phosphorylated and enters the nucleus to regulate the downstream transcription factor Daf-16, thereby prolonging the lifespan. When the inactivation of the upstream kinase in the insulin signaling pathway causes it to transfer from the cytoplasm to the nucleus, three conditions occur: cytoplasm, nucleus, and intermediate position (Henderson and Johnson, 2001). The L4 TJ356 nematodes were treated with different concentrations of SVHRSP for 10 consecutive days. Each group of 20–30 TJ356 nematodes with different concentrations of SVHRSP was washed with M9 solution at least three times. The next day, the nematodes were transferred to the fresh NGM plate containing different concentrations of SVHRSP; 20–30 nematodes with different concentrations of SVHRSP were selected and covered with another slide in parallel. The AGAR was flattened to prevent breakage, and bubbles were removed. The nematodes were placed under an inverted fluorescence microscope for examination and statistical fluorescence intensity. The excitation wavelength is 488 nm, and the emission wavelength is 500–530 nm. Image J software was used to analyze the fluorescence intensity, and the nucleation experiment was repeated at least three times.

### Fluorescence Measurements in the CF1553 Strain and the TJ375 Strain

The CF1553 nematodes carrying Sod-3-GFP and the TJ375 nematodes carrying Hsp-16.2-GFP were imaged using fluorescence microscope (Leica DM4000B). Two kinds of nematodes were treated with the same lifespan experiment. During imaging, the excitation wavelength was 470 nm, and GFP was recorded at 550 nm. The total fluorescence of each worm was analyzed using ImageJ software.

### Experimental Statistical Analysis

GraphPad Prism 9.0 was used for experimental statistical analysis. For life tests, Kaplan–Meier survival was used, and *p*-values were calculated using log-rank tests. One-way analysis of variance (ANOVA) with Duncan’s test was used multigroup comparisons of other experiments. A *p-*value of <0.05 was considered statistically significant.

## Results

### Scorpion Venom Heat–Resistant Synthesized Peptide Extends the Lifespan and Reduced the Level of Lipofuscin Without Affecting the Normal Physiological Function in *Caenorhabditis elegans*

**FIGURE 1:**
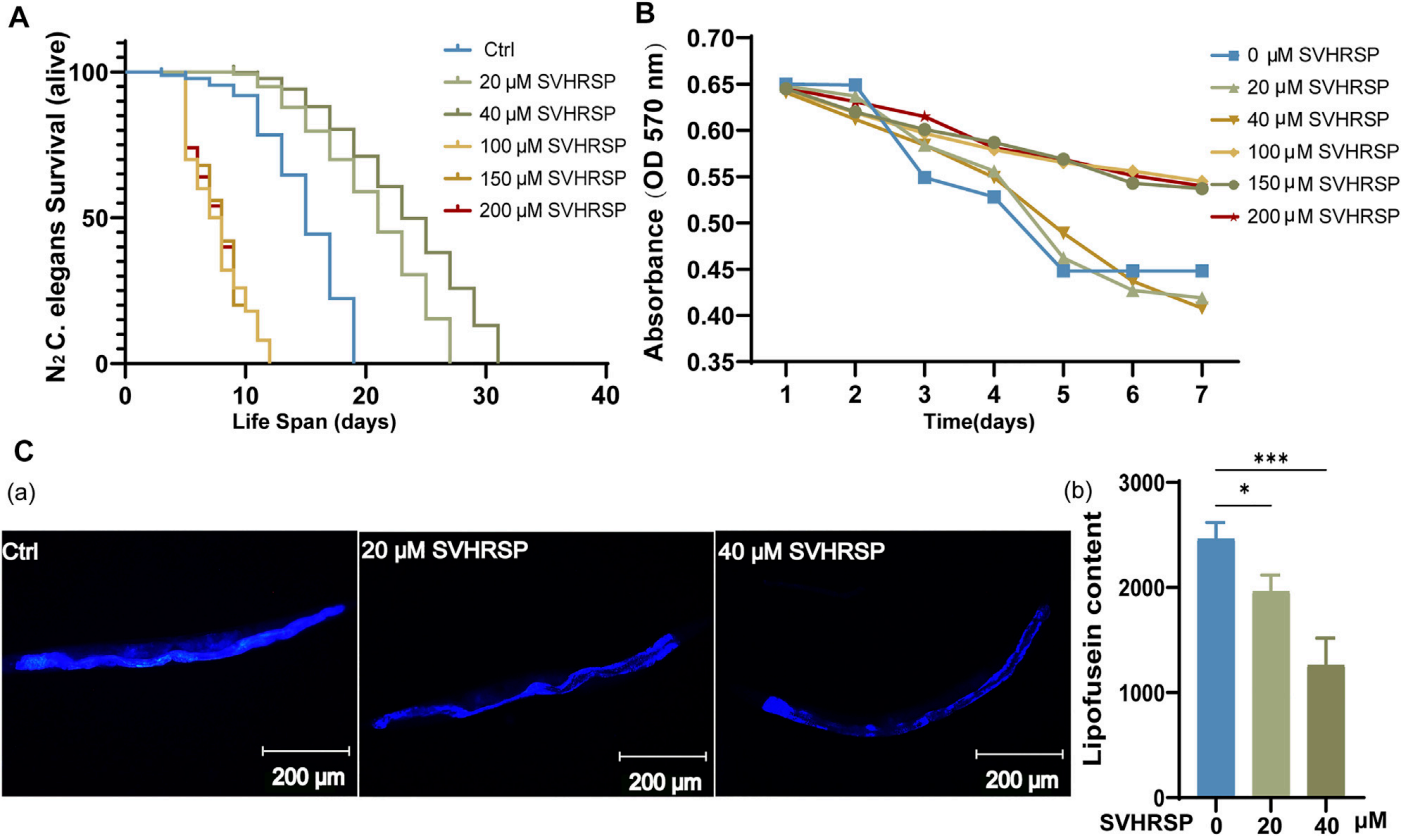
Scorpion venom heat–resistant synthesized peptide (SVHRSP) extends the lifespan of C. *elegans*. **(A)** survival curve of *C. elegans* N_2_ at 20°C treatment with 20-, 40-, 100-, 150-, or 200-μmol/L SVHRSP or with ddH_2_O (control) starting from the L4 stage (day 0), the survival number of worms in each group was recorded until all worms died; 40-μMol/L SVHRSP appeared to extend the longest lifespans (***p < 0.001, log-rank test). **(B)** effects of SVHRSP on food intake in N_2_ worms. Surplus *E. coli* OP50 was detected by OD 600. Data were analyzed by ordinary one-way analysis of variance (ANOVA) using Prism 9.0. Values are presented as mean ± SEM. *p < 0.05. **(C)** representative images of C. *elegans* showing the accumulation of autofluorescent lipofuscin treatment with SVHRSP or not. The scale bar was 200 μm. Total fluorescence per worm was analyzed using ImageJ software. Data were analyzed by ordinary one-way ANOVA using Prism 9.0. Values are presented as mean ± SEM; 20-μM SVHRSP: *p < 0.05; 40-μM SVHRSP: ***p < 0.001.

To evaluate whether SVHRSP can prolong worm lifespan and screen out the optimal drug concentration, we first treated wild-type N_2_ worms with SVHRSP at a concentration of 0–200 μM on an NGM plate. The results indicate that when the concentration of SVHRSP was greater than 100 μM, the average life span of nematodes was significantly shortened, indicating that100 μM or higher concentrations of SVHRSP had significant impairment on the health status of wild-type N_2_ worms (Figure 1A) (*p* < 0.001). In a similar way, we investigated the effects of lower doses (20-and 40-μM SVHRSP) on the worms. We discovered that the wild-type N_2_ worms, when treated with ddH_2_O (control) and doses of 20-and 40-μM SVHRSP, would survive. Posttreatment with a low dose of SVHRSP, the worm’s lifespan was significantly prolonged (Figure 1A) (*p* < 0.001). These results indicate that SVHRSP can prolong nematode life span and has a dose–effect relationship. The results of the food intake test showed that SVHRSP at 100, 150, and 200 μM affected the feeding activity of nematodes, while SVHRSP at 20 and 40 μM had no effect on feeding activity of nematodes (Figure 1B) (*p* < 0.05). Therefore, 20- and 40-μM SVHRSP concentrations could be used as effective doses for subsequent experiments. As the growth of nematode age progresses, the level of lipofuscin in the nematode body keeps rising (Henderson and Johnson, 2001). This is a normal physiological phenomenon associated with the nematode body. Aging is usually accompanied by the emergence of highly oxidized and crosslinked proteins. These oxidized protein aggregates are lipofuscin. Because protease or lysosome cannot degrade lipofuscin, it is regarded as the “marker dye” of aging (Wang H et al., 2018). On the 10th day of administration, we tested the effect of 20- and 40-μM SVHRSP on lipofuscin accumulation in aged *C. elegans*. We discover that 20- and 40-μM SVHRSP can significantly reduce the level of lipofuscin in aging nematodes (Figures 1A–C) (*p* < 0.05, *p* < 0.001). This provides direct evidence to support the antiaging ability of SVHRSP. In conclusion, 20- and 40-μM SVHRSP can prolong the life and reduce the level of lipofuscin in nematodes. In order to know whether SVHRSP only improved lifespan or it also had positive effects on healthspan, we need to investigate further. Thus, we evaluated the effects of 20- and 40-μM SVHRSP studies on age-related physiological functions, including pharyngeal pumps, body bending, food intake, and spawning numbers. During aging, muscle cells throughout the body gradually lose their vitality, resulting in decreased activity (Ryu et al., 2016). Reduced motor capacity is the most prominent clinical feature of aging. The rhythmic beating of muscles in the pharyngeal region of *C. elegans* back of the head is called the pharyngeal pump. Aging and neuronal injury can cause the pharyngeal beating of *C. elegans* to slow down. Therefore, the pharyngeal pump rate can be selected as an indicator to evaluate the health of *C. elegans* and the efficacy of drugs (Ryu et al., 2016). We tested the changes in the pharyngeal pump and body-bending rate of nematodes treated with 20- and 40-μM SVHRSP. The results show that SVHRSP did not enhance body bending and pharyngeal pumping contraction in worms (Supplementary Figures S1A,B) (*p* > 0.05). The results showed that SVHRSP could not improve the locomotor capacity of nematodes caused by aging and did not improve the feeding ability of nematodes. This suggests that exercise and dietary restriction (DR) may not be one of the reasons for the antiaging mechanism of SVHRSP. One of the most popular theories of aging is longevity and reproduction. Using a variety of advanced animal models of aging, there were reports of a mechanism called a “tradeoff” between longevity and fertility, in which reduced or lost fertility leads to increased longevity (Gruber et al., 2007). However, in this study, SVHRSP did not affect the oviposition rate, suggesting that SVHRSP prolonged the lifespan of nematodes and did not lead to a reduction in nematodes’ reproductive capacity. (Supplementary Figure S1C) (*p* > 0.05). These results indicate that SVHRSP does not affect the normal physiological functions of nematodes in the process of prolonging their lifespan.

### Scorpion Venom Heat–Resistant Synthesized Peptide Increased Stress Resistance of *Caenorhabditis elegans*

**FIGURE 2:**
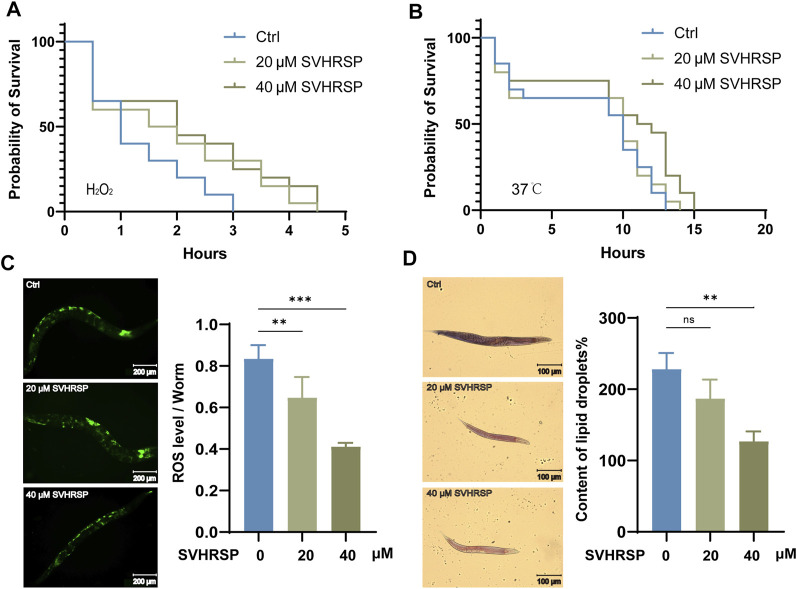
Scorpion venom heat–resistant synthesized peptide (SVHRSP) significantly increased the resistance of nematodes to stress. **(A)** mean survival time of wild-type worms treated with 20- and 40-μM SVHRSP at 30% H_2_O_2_. Log-rank verifies the calculated *p*-value. *p < 0.05 **(B)** the mean survival time of wild-type worms treated with 20- and 40-μM SVHRSP at 37°C. Log-rank verifies the calculated *p*-value. *p < 0.05 **(C)** effects of 20- and 40-μM SVHRSP on reactive oxygen species levels in wild-type worms. **(D)** the effect of SVHRSP on fat accumulation. SVHRSP significantly inhibited the accumulation of excess fat in N_2_ nematodes. Values are presented as mean ± SEM. All of these measurements were made at least three times. Log-rank and one-way analysis of variance verifies the calculated *p*-value. ns means none significant; *p < 0.05; **p < 0.01; ***p < 0.001.

As worms age, their resistance to external stimuli declines sharply, and the increase in endogenous ROS production accelerates the aging process further, thereby creating a vicious cycle. (Harman, 1956). In order to study whether SVHRSP can improve the stress resistance of nematodes, nematodes were treated with 20- and 40-μM SVHRSP. This study examined whether SVHRSP could increase the resistance of N_2_ wild-type senescence *C. elegans* to acute oxidative stress and acute heat stress. On the 10th day of administration, 30% H_2_O_2_ was used to induce acute oxidative stress in nematodes, heat stress was induced at 37°C, and the survival time of nematodes was recorded. We discovered that SVHRSP pretreatment had a significant protective effect on these stress injuries (Figures 2A,B) (*p* < 0.05), suggesting that SVHRSP enhanced the resistance of nematodes to oxidative and thermal stress. Normal metabolic activity of cells produces a large accumulation of oxygen free radicals (ROS), and it has been reported that high levels of ROS can lead to aging (Harman, 1956). ROS levels in nematodes could be quantified using H_2_DCF-DA fluorescent probe. In this experiment, the ROS level of N_2_ wild-type senescence *C. elegans* was detected for different concentrations of SVHRSP. Our results showed that ROS accumulation was reduced in SVHRSP-pretreated worms (Figure 2C) (20-μM SVHRSP: *p* < 0.005, 40-μM SVHRSP: *p* < 0.001), suggesting that SVHRSP may prolong worm lifespan by reducing ROS levels in worms. It may be related to the increase in SOD activity in aged nematodes. In wild-type worms, increased ROS lead to excessive accumulation of fat (Hughes et al., 2007). Considering that SVHRSP can effectively reduce ROS levels in nematodes. SVHRSP pretreatment and untreated N_2_ worms were stained with oil red O to study the effect of SVHRSP on fat deposition. The results showed that 40-μM SVHRSP significantly reduced fat accumulation in nematodes (Figure 2D) (*p* < 0.01). These results suggest that SVHRSP can reduce lipid accumulation caused by ROS production.

### Scorpion Venom Heat–Resistant Synthesized Peptide-Mediated Longevity Extension is Associated with the Insulin/IGF-1 Signaling Pathway

**FIGURE 3:**
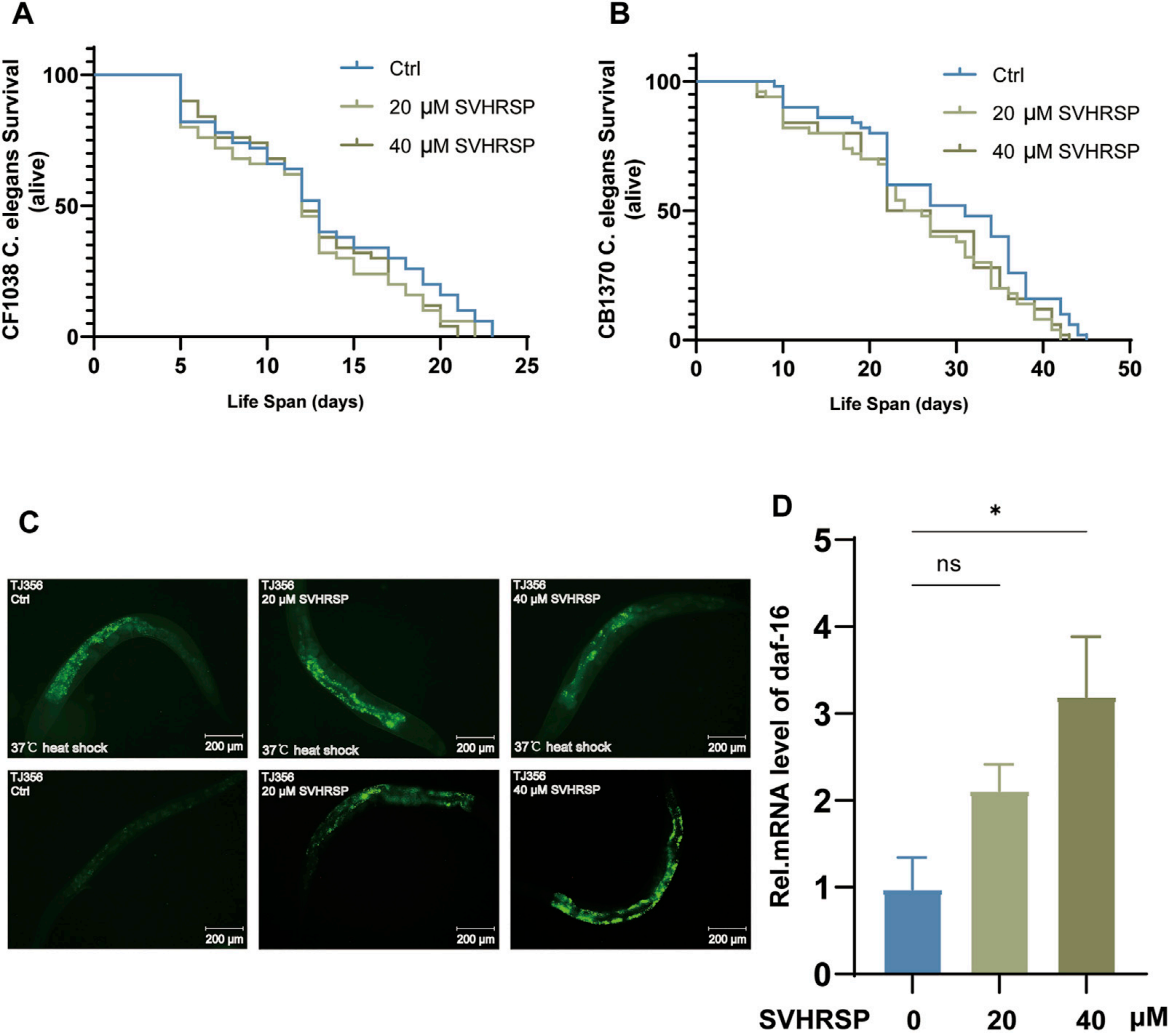
Scorpion venom heat–resistant synthesized peptide (SVHRSP)-mediated longevity effects depended on the insulin/IGF-1-like signal (IIS) pathway. **(A)** effects of 20- and 40-μM SVHRSP untreated treatments on the lifespan of CF1038 nematodes. **(B)** effects of 20- and 40-μM SVHRSP treatments on the lifespan of CB1370 nematodes. **(C)** Fluorescence images of TJ356 nematodes treated with 20-μM and 40-μM SVHRSP, and untreated under heat stress. **(D)** Daf-16 mRNA expression levels of wild-type nematodes treated with 20- and 40-μM SVHRSP untreated. Values are presented as mean ± SEM. All of these measurements were made at least three times. Log-rank and one-way analysis of variance verifies the calculated *p*-value. ns means none significant; *p < 0.05; **p < 0.01; ***p < 0.001.

According to the above experiments, SVHRSP has an excellent performance in nematodes’ resistance to stress, and the insulin/insulin-like growth factor-1 signaling pathway (IIS pathway) is the most widely studied senescence-related pathway, which is related to stress, metabolism, growth, longevity, and behavior (Murphy and Hu, 2013). Daf-2 controls the activity of the conserved-phosphatidylinositol 3-kinase (PI3K)/Akt kinase cascade, which ultimately regulates the FOXO transcription factor Daf-16 (Tullet, 2015). The transcription factor controls most of the functions of this pathway and its downstream regulation of Sod-3, Hsp-16.2, Ctl-1, Gei-7, and other stress-related genes. To determine whether SVHRSP extends lifespan through the IIS pathway, we performed lifespan tests using the Daf-2 (E1370) mutant nematode CB1370 and Daf-16 (Mu86) mutant nematode CF1038 mutants *C. elegans*. The results showed that neither 20-- nor 40-μM SVHRSP could prolong the life span of CF1038 and CB1370, suggesting that Daf-2 and Daf-16 may be necessary for SVHRSP to prolong the life span of *C. elegans* (Figures 3A,B). (*p* > 0.05). Since Daf-16 plays an important role in the IIS pathway, the effect of SVHRSP on Daf-16 expression was further confirmed. We used transgenic nematode TJ356 ZIS356 IV to investigate whether SVHRSP could affect the expression of Daf-16. The Daf-16 gene of the nematode was labeled with GFP. An increase in culture temperature accelerates DAF‐16 nuclear accumulation. When *C. elegans* was at 37°C, the amount of Daf-16 was increased by heat stress. SVHRSP can reduce the damage of this stress state to *C. elegans* and reduce the amount of nuclear entry. It alleviates the stress state of the *C. elegans* itself. However, when *C. elegans* were in a normal aging state, the expression of Daf-16 was impaired, and the intake of SVHRSP improved the health status of *C. elegans* and promoted the expression of DAF-16. The results show that SVHRSP can reduce the nuclear displacement of Daf-16 at 20 and 40 μM under heat shock at 37°C, and the nuclear translocation level of TJ356 nematode was increased on day 10 of administration. (Figure 3C). In addition, N_2_ wild-type *C. elegans* were collected on day 10 of administration, and RNA was extracted for qPCR test to detect the change in Daf-16 mRNA level, with act-1 as the steward gene. Real-time quantitative PCR results showed that 40-μM SVHRSP–treated worms had an increased mRNA level of Daf-16 compared with untreated control (Figure 3D). In conclusion, the effect of SVHRSP on senescence in nematodes may be mediated using the IIS pathway.

### Scorpion Venom Heat–Resistant Synthesized Peptide Regulates Downstream Gene Expression of IIS Pathway

**FIGURE 4:**
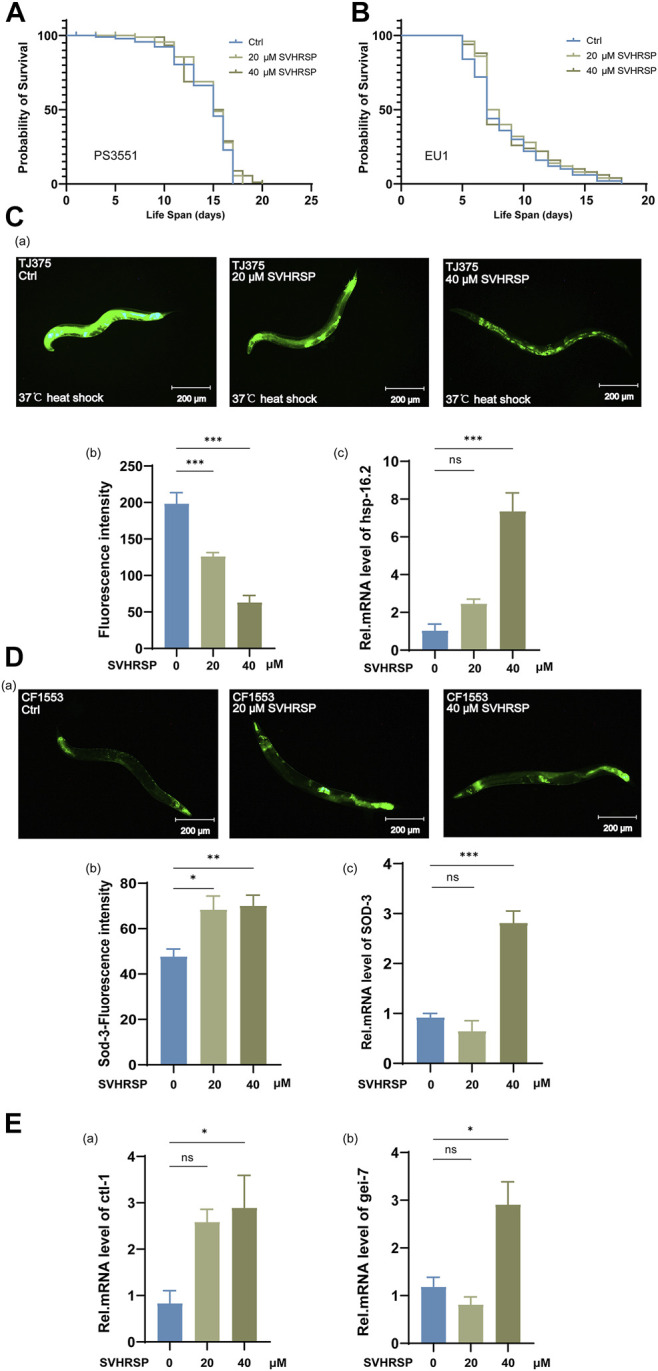
Scorpion venom heat–resistant synthesized peptide (SVHRSP) regulates downstream gene expression of the IIS pathway. **(A)** effects of 20- and 40-μM SVHRSP untreated treatments on the lifespan of PS3551 nematodes. **(B)** -effects of 20- and 40-μM SVHRSP untreated treatments on the lifespan of EU1 nematodes. **(C) (a)** fluorescence images of TJ375 nematodes treated with 20- and 40-μM SVHRSP, and untreated under heat stress. **(b)** the GFP strength from the TJ375 strain was treated with 20- and 40-μM SVHRSP. **(c)** Hsp-16.2 mRNA levels in worms treated with 20- and 40-μM SVHRSP and controls. **(D) (a)** fluorescence images of CF1553 nematodes treated with 20- and 40-μM SVHRSP, and untreated. **(b)** the GFP intensity of CF1553(muIs84) strain after 20- and 40-μM SVHRSP treatment was determined. **(c)** Sod-3mRNA levels in worms treated with 20- and 40-μM SVHRSP and controls. **(E) (a)** Ctl-1 mRNA levels in worms treated with 20- and 40-μM SVHRSP and controls. **(b)** Gei-7 mRNA levels in worms treated with 20- and 40-μM SVHRSP and controls. Values are presented as mean ± SEM. Log-rank and one-way ANOVA. ns means none significant; *p < 0.05; **p < 0.01; ***p < 0.001. All of these measurements were made at least three times.

The above experiments prove that SVHRSP may be related to the IIS pathway. Moreover, SVHRSP showed excellent performance in helping *C. elegans* resist stress. Therefore, we investigated the effect of SVHRSP on downstream Daf-16 related genes. Hsf-1 is an important long-lived transcription factor that acts downstream of the IIS and is widely studied. It has been reported that downregulation of the IIS pathway, along with coactivation of Hsf-1, is necessary for the prolongation of life (Chauve et al., 2021). To investigate whether Hsf-1 is required for SVHRSP-mediated life extension, we tested the effects of 20- and 40-μM SVHRSP on Hsf-1 (SY441) mutant worms PS3551. The results showed that SVHRSP did not prolong the lifespan of Hsf-1 mutant worms PS3551 (Figure 4A) (*p* > 0.05), suggesting that Hsf-1 is indeed necessary for its lifespan extension.

Skn-1 is a lineal of human NRF/CNC (nuclear factor E2-related factor 2, NRF2; Capncollar, CNC) protein and plays an important role in stress response and homeostasis Skn-1 promotes longevity in otherwise WT animals (Blackwell et al., 2015). Because of SVHRSP’s excellent performance in helping *C. elegans* resist stress. We used Skn-1 (ZU67) mutant *C. elegans* EU1 to conduct life span experiments. The results showed Skn-1 (ZU67) mutant’s worms EU1, treated with 20- and 40-μM SVHRSP, failed to extend the lifespan when compared to ddH_2_O control group (Figure 4B) (*p* > 0.05), suggesting that Skn-1was also required for its lifespan extension.

Our previous experiments have shown that SVHRSP enhances heat stress resistance in nematodes (Figure 2B). To determine whether this is related to SVHRSP regulating specific stress response genes, we used transgenic *C. elegans* TJ375 (gpIs1) (Hsp-16.2p:GFP); the Hsp-16.2 gene of the nematode was labeled with green fluorescent protein (GFP). Inducible GFP fluorescence after >1 h heat shock at 35°C. On day 10 of administration, 20- and 40-μM SVHRSP decreased Hsp-16.2 expression in TJ375 nematodes under heat stress (Figures 4A–C) (20-μM SVHRSP: *p* < 0.05, 40-μM SVHRSP: *p* < 0.001). These results indicated that Hsp-16.2 could be overexpressed in *C. elegans* under heat stress, and SVHRSP could reduce the expression of Hsp-16.2. These results indicated that SVHRSP might have a protective effect on the heat stress of nematodes. In addition, N_2_ wild-type *C. elegans* were collected on day 10 of administration, and RNA was extracted for qPCR test to detect the change in Hsp-16.2 mRNA level, with act-1 as the steward gene. Real-time quantitative PCR results showed that the expression of Hsp-16.2 was significantly increased after 10 days of treatment with 20- and 40-μM SVHRSP (Figure 4C) (40-μM SVHRSP *p* < 0.01). These results indicate that SVHRSP can increase the mRNA level of Hsp-16.2 in aging nematodes, suggesting that SVHRSP may help nematodes resist stress and delay aging by increasing the expression level of heat stress protein Hsp-16.2 in nematodes.

Considering the antioxidant effect of SVHRSP in nematodes, we studied whether the downstream gene Sod-3 of the IIS pathway has an effect on worm lifespan and stress resistance. Sod-3 has the activity of protein homodimerization and superoxide dismutase (Sod). It participates in the removal of superoxide free radicals in nematodes (Sheng et al., 2014). In order to detect the effect of SVHRSP on the expression of Sod-3 in *C. elegans*, we use the genetically-modified nematode CF1553, (muIs84) (SOD-3p:GFP + ROL-6). The Sod-3 gene of the nematode was labeled with a GFP. After 10 days of treatment with 20- and 40-μM SVHRSP, the expression level of Sod-3 in earthworms was higher than that in the control group (Figures 4A,B,D) (20-μM SVHRSP: *p* < 0.05; 40-μM SVHRSP: *p* < 0.01). In addition, N_2_ wild-type *C. elegans* were collected on day 10 of administration, and RNA was extracted for qPCR test to detect the change in Sod-3 mRNA level, with act-1 as the steward gene. The results of real-time quantitative PCR showed that the expression of Sod-3 in nematodes treated with 20- and 40-μM SVHRSP for 10 d was significantly increased compared with the untreated group (Figures 4C,D). (40-μM SVHRSP: *p* < 0.01). These results suggest that SVHRSP can increase the expression of Sod-3 in nematodes, suggesting that nematodes may resist reactive oxygen generation by increasing the expression level of SOD in aging nematodes, thus maintaining homeostasis and delaying senescence.

We further investigated whether the effect of SVHRSP on lifespan was related to additional age-related genes and examined the transcriptional levels of Ctl-1 and Gei-7. Ctl-1 and Gei-7 enable catalase activity (Taub et al., 2003). It also has been predicted to enable isocitrate lyase activity and malate synthase activity, along with reports indicating significant involvement in the determination of adult lifespan (Gallo et al., 2011). Therefore, N_2_ wild-type *C. elegans* were collected on day 10 of administration, and RNA was extracted for qPCR test to detect the change in Ctl-1 and Gei-7 mRNA levels, with act-1 as the steward gene. We found that the mRNA levels of Ctl-1 and Gei-7 were significantly increased after SVHRSP treatment (Figures 4A,B,E) (40-μM SVHRSP: *p* < 0.05). These results suggest that the effect of SVHRSP on prolonging life and improving antistress effects are partly dependent on the expression of stress-induced genes.

## Discussion

With increased age, the function of each component of the body decreases, and along with aging, the incidence of age-related diseases also increases. Therefore, the search for drugs that can prevent or delay aging has attracted more and more attention. More than 60% of all medicines currently on the market are related to the structure and information of natural products, so natural products are an important source of new drugs in the drug discovery process. Scorpion venom, containing a variety of toxin peptides, has diverse biological activities and is used as a medicine to strengthen human health. As an active peptide extracted from the Buthusmartensii Karsch venom, previous studies from our research group had shown that SVHRP exhibited many beneficial neuroprotective effects (Wang et al., 2007; Chen et al., 2021). SVHRSP is a synthesized peptide according to the amino acid sequence of SVHRP. In line with previous findings (Xu et al., 2021), SVHRSP alleviated neuroinflammation by downregulating microglial Nav1.6 to protect dopamine neurons (Li et al., 2021). So, we predict SVHRSP should beneficially affect lifespan.

In this study, we used *C. elegans* as a model to evaluate the effects of SVHRSP on the longevity of senescent nematodes and their related mechanisms.

In our study, we first confirmed whether SVHRSP could prolong the life span of *C. elegans*. The results showed that SVHRSP was not toxic and extended the lifespan of worms at certain concentrations (20 µM and 40 µM). As *C. elegans* age growing, *C. elegans* fat brown levels rise. This is the inevitable phenomenon of nematode growth. Lipofuscin is produced by the body’s normal physiological metabolism in the aging process of highly oxidized protein aggregation because it accumulates in the body as a nematode worm signature dye (Martins et al., 2017). Our results found that supplementation with SVHRSP not only significantly extended the lifespan of *C. elegans* but also reduced the accumulation of age lipofuscin, consistent with many other natural extracts and bioactive compounds involved, such as Paullinia cupana (Boasquívis et al., 2018), Momordica charantia (Cao et al., 2018), and Polygonum multiflorum (Büchter et al., 2015), providing direct evidence to support the general antiaging capability of SVHRSP. The process of aging is usually accompanied by physiological changes, such as the gradual loss of muscle cell vitality during aging, resulting in a decrease in the motility of nematodes (Murakami et al., 2005). Pharyngeal pumping rate decreased with age, reflecting a decrease in nematode food intake and inducing DR effects (Ryu et al., 2016; Herndon et al., 2018). Many studies of natural products have demonstrated that antiaging effects improve these indicators (Fang et al., 2017; Lin et al., 2019). In addition, the relationship between longevity and fertility has been a hot topic in many theories of aging. In studies of various animal models, scientists have made an interesting discovery that reduced fertility may lead to increased longevity. (Herndon et al., 2018). Therefore, we investigated reproductive parameters, such as oviposition volume, pharyngeal pumping rate, and body-bending ability of nematodes, to reflect the effects of oligopeptide treatment on age-related physiological indexes of nematodes (Yang et al., 2018). In this study, we found that SVHRSP did not improve the motility or pharyngeal pump rate of nematodes. These results indicated that SVHRSP could prolong the lifespan of nematodes without affecting their reproductive ability. In conclusion, the effects of exercise and DR may not be the main reasons behind SVHRSP-mediated antiaging related mechanisms, suggesting that SVHRSP extended the lifespan and reduced the level of lipofuscin without affecting the normal physiological function in *C. elegans*.

Oxidative stress is a pathological state related to aging. A large number of studies have shown that oxidative stress will cause certain damage to the body (Avanesov et al., 2014; Beckhauser et al., 2016). *C. elegans*’ longevity is associated with increased resistance to external stimuli (Morley and Morimoto, 2004; Westerheide et al., 2009; Fontana et al., 2010; Schieber and Chandel, 2014). To ensure the beneficial effects of SVHRSP on nematode survival, we examined its resistance to heat and oxidative stress. The results showed that SVHRSP could enhance the resistance of nematodes to external stimuli. The free radical theory of aging is a widely accepted hypothesis that describes ROS as the key to aging and age-related diseases and that the accumulation of excessive ROS in nematodes greatly shortens the lifespan of nematodes (Avanesov et al., 2014). Nematodes normally produce large amounts of antioxidant enzymes, such as Sod, to fight ROS production and maintain balance (Wang et al., 2016). Our results suggest that SVHRSP reduces ROS accumulation in nematodes. Growing evidence has shown that enhanced ROS production can lead to excessive fat accumulation and change in fatty acid composition, at least in part depending on the Daf-16 pathway (Brunk and Terman, 2002; Hughes et al., 2007; Wang K et al., 2018). After lipid staining with oil red O, the results showed that SVHRSP could reduce fat accumulation and promote lipid metabolism in *C. elegans*. This is consistent with the results of previous ROS studies. It is implied in this study that SVHRSP enhanced tolerance to heat stress and H_2_O_2_-induced oxidative stress in *C. elegans* and effectively decreased ROS levels and fat accumulation levels, which are in accordance with many other antioxidants, such as flavonoids (Yang et al., 2019), Quercetin (Proshkina et al., 2016), and Naringin (Zhu et al., 2020). In conclusion, SVHRSP may play an important role in the regulation of redox balance in nematodes, which may contribute to the study of its antiaging mechanism.

The IIS pathway is an important pathway regulating aging and longevity and is related to stress, metabolism, growth, and longevity behavior (Fontana et al., 2010; Kenyon, 2010). It is highly conserved in invertebrates and mammals and is one of the most well-understood and widely studied senescence-related pathways (Panowski and Dillin, 2009). Daf-2 is a growth factor receptor of the IIS pathway, which plays a major role in nematode growth and metabolism. With the growth of nematodes, Daf-2 can downregulate the metabolic activity of nematodes. Therefore, the loss of Daf-2 can prolong the lifespan of the nematode and enhance its stress resistance, and its downregulation can promote the expression of Daf-16 downstream (Murphy et al., 2003; Rea et al., 2005; Sun et al., 2017). FOXO homolog Daf-16 of *C. elegans* is the core transcription target of the IIS pathway, which plays a positive role in regulating lifespan, stress, reproduction, lipid metabolism, and other biological processes in IIS. Its destruction reportedly speeds up the normal rate of aging, and boosting its expression can extend its lifespan (Garigan et al., 2002; Lee et al., 2003; Haithcock et al., 2005). Our results showed that SVHRSP could not prolong the life span of Daf-2 and Daf-16 mutant nematodes. These findings suggest that Daf-2 and Daf-16 may be key molecules necessary for SVHRSP to prolong the lifespan of nematodes. In another study in our lab, we used PD models of 6-OHDA-induced nematodes. The lifespan of CB1370 nematodes was significantly shortened after induction with 6-OHDA but recovered after treatment with SVHRSP (not published yet). These results suggest that the delayed life of SVHRSP may be related to Daf-2. In the following experiments, we will order daf-2 overexpressed nematodes with gene mutation for the next experiment to determine the relationship between SVHRSP and Daf-2. Considering that the IIS pathway regulates lifespan in *C. elegans* through Daf-16 nuclear localization (Narasimhan et al., 2011), we postulated that SVHRSP treatment might affect Daf-16 nuclear translocation, a necessary prerequisite for its transcriptional activation activity. Using the nematode labeled Daf-16 gene with GFP, we found SVHRSP can reduce the nuclear displacement of Daf-16 under heat shock at 37°C, and the nuclear translocation level of the TJ356 nematode was increased on day 10 of administration. And meanwhile, the expression level of Daf-16 mRNA was increased by SVHRSP in N_2_ wild-type *C. elegans*.

Heat shock factor 1 (Hsf-1) regulates the expression of cellular chaperone genes to maintain the proteostasis from stresses by preventing misfolding and mistranslation (Hsu et al., 2003; Chiang et al., 2012) and regulates the expression of the IIS pathway downstream genes synergizing with Daf-16. It has been reported that overexpression of Hsf-1 results in increased longevity, whereas inhibition of Hsf-1 activity by RNAi reduces lifespan in mammals (DanQing et al., 2021). In the present research, we subsequently used worms lacking functional Hsf-1 genes, Hsf-1 (sy441), for the lifespan assays and found that SVHRSP treatment indeed failed to extend the lifespan of Hsf-1 (sy441) worms. These results implied that Hsf-1 was required for SVHRSP-mediated induced heat resistance and lifespan extension of *C. elegans*. It has been reported that baicalein mediates antioxidant effects by activating nuclear factor erythroid 2-related factor 2 (Nrf-2) in mammalian cell lines (Ma J et al., 2018). When it comes to *C. elegans*, Skn-1, homologous to mammalian Nrf-2 (An and Blackwell, 2003), is known as a major regulator of longevity and oxidative stress responses through the IIS pathway (Lee et al., 2015; Altintas et al., 2016; Tullet et al., 2017). Skn-1 has been proven to improve lifespan and ease Aβ-induced toxicity in nematodes (Edwards et al., 2014; Govindan et al., 2018). Further analysis revealed that SVHRSP failed to extend the lifespan of mutant worms for Skn-1. These results implied that Skn-1 was required for SVHRSP-mediated induced heat resistance and lifespan extension of *C. elegans*. Hsp-16.2, a downstream target gene of the IIS pathway, is known to be regulated by Hsf-1 and associated with aging and many age-related diseases (Leroux et al., 1997). Hsp-16.2 can also downregulate oxidative stress by increasing the levels of the reduced form of glutathione (Mehlen et al., 1996) and modulate longevity by interacting with nuclear hormone receptors (Morimoto, 2002). Our results also provide evidence that Hsp-16.2 could be overexpressed in *C. elegans* under heat stress, and SVHRSP could reduce the expression of Hsp-16.2. In addition, we performed a qPCR test on N_2_ *C. elegans* on day 10 of administration and found that SVHRSP can increase the mRNA level of Hsp-16.2 in aging nematodes, suggesting that SVHRSP may help nematodes resist stress and delay aging by increasing the expression level of heat stress protein Hsp-16.2 in nematodes.

As a direct target of Daf-16, Sod-3 is a typical scavenger enzyme that protects the worms against ROS and declines with aging (Rangsinth et al., 2019). Its promoter contains consensus Daf-16/FOXO-binding elements (DBEs), which bind to Daf-16 and catalyze the removal of superoxide to resist oxidative stress (Oh et al., 2006). We found that SVHRSP could slow down the decline of the mRNA expression of Sod-3 with aging in worms. For further verification of the effect of SVHRSP, we analyzed the transgenic strain CF1553, carrying a Sod-3-GFP reporter gene, and found that Sod-3-GFP showed higher expression levels treated with SVHRSP, which might exert the antiaging effects of SVHRSP *via* the increasing activity of antioxidant enzymes. To this end, we detected changes in Sod-3 mRNA expression level of N_2_ nematodes on day 10 of administration. We found that SVHRSP can increase the expression of Sod-3 mRNA in worms. Afterward, we also detected the expression levels of Ctl-1 and Gei-7 downstream stress-related genes of Daf-16. The results showed Ctl-1 and Gei-7, as antioxidant genes related to the IIS pathway, were also upregulated following SVHRSP treatments at the mRNA level and might be contributing to the antioxidant activity of SVHRSP. These results suggest that SVHRSP may delay aging by affecting downstream stress-related target genes through Daf-16.

## Conclusion

Through the above experimental studies, we found that SVHRSP significantly prolonged the life span and delayed aging of nematodes through insulin/IGF-1 signal transduction and stress resistance pathways. These pathways are well conserved from worms to mammals. Our results can be the basis for developing SVHRSP-rich products with health benefits, and provide a platform for further research on the age-related or progressive diseases in mammals.

## Data Availability

The raw data supporting the conclusions of this article will be made available by the authors without undue reservation.
