# Supplementary figures and images for: Scorpion Venom Heat–Resistant Synthesized Peptide Increases Stress Resistance and Extends the Lifespan of Caenorhabditis elegans via the Insulin/IGF-1-Like Signal Pathway
Source: Front Pharmacol. 2022 Jul 14;13:919269. doi: 10.3389/fphar.2022.919269 (PMC9330001; doi:10.3389/fphar.2022.919269)

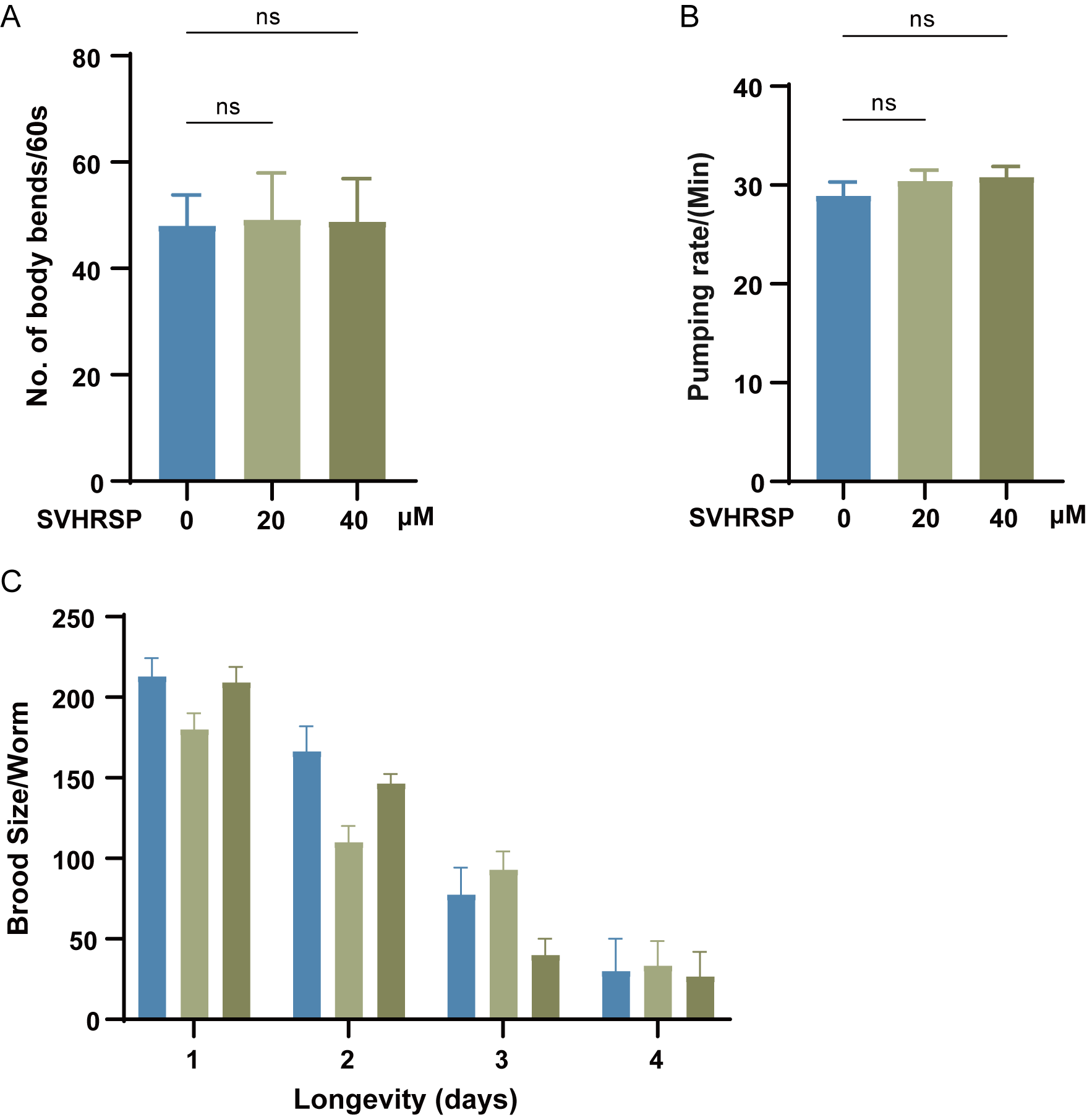

Supplement: Supplementary file 1 [file Image1.tif]
